# Revealing the impact of TOX3 on osteoarthritis: insights from bioinformatics

**DOI:** 10.3389/fmed.2023.1256654

**Published:** 2023-11-08

**Authors:** Zhengyan Wang, Shuang Ding, Chunyan Zhang, Hongsheng Zhan, Yunfei Li, Jing Yan, Yuyan Jia, Xukai Wang, Ying Wang

**Affiliations:** ^1^College of Traditional Chinese Medicine, Changchun University of Chinese Medicine, Changchun, China; ^2^Department of Orthopedics, The Affiliated Hospital of Changchun University of Chinese Medicine, Changchun, China; ^3^General Ward, Jilin Cancer Hospital, Jilin, China; ^4^Department of Orthopedics, Shuguang Hospital, Shanghai, China

**Keywords:** osteoarthritis, TOX high mobility group box family member 3, machine learning models, immune infiltration analysis, ceRNA network

## Abstract

Osteoarthritis, a prevalent long-term condition of the joints, primarily impacts older individuals, resulting in discomfort, restrictions in mobility, and a decrease in overall well-being. Although Osteoarthritis is widely spread, there is a lack of successful interventions to stop the advancement of the condition. Numerous signaling pathways have been emphasized in recent research on Osteoarthritis, yet the diagnostic significance of numerous genes has not been investigated. To identify genes that were expressed differently in osteoarthritis, we utilized the Gene Expression Omnibus database. To identify marker genes, we built machine learning models including Least Absolute Shrinkage and Selection Operator and Random Forest. We categorized Osteoarthritis samples and performed immune cell infiltration analysis based on the expression patterns of these characteristic genes. Both the Least Absolute Shrinkage and Selection Operator and Random Forest models selected six marker genes (TOX3, ARG1, CST7, RERGL, COL11A1, NCRNA00185) out of a total of 17 differentially expressed genes. The osteoarthritis samples were categorized into two groups, namely a high expression group and a low expression group, based on the median levels of TOX3 expression. Comparative analysis of these groups identified 85 differentially expressed genes, showing notable enrichment in pathways related to lipid metabolism in the group with high expression. Analysis of immune cell infiltration revealed noticeable differences in immune profiles among the two groups. The group with high expression of TOX3 showed a notable increase in Mast cells and Type II IFN Response, whereas B cells, Cytolytic activity, Inflammation-promoting cells, NK cells, pDCs, T cell co-inhibition, Th1 cells, and Th2 cells were significantly decreased. We constructed a ceRNA network for TOX3, revealing 57 lncRNAs and 18 miRNAs involved in 57 lncRNA-miRNA interactions, and 18 miRNA-mRNA interactions with TOX3. Validation of TOX3 expression was confirmed using an external dataset (GSE29746), revealing a notable increase in Osteoarthritis samples. In conclusion, our study presents a comprehensive analysis identifying TOX3 as a potential feature gene in Osteoarthritis. The distinct immune profiles and involvement in fat metabolism pathways associated with TOX3 expression suggest its significance in Osteoarthritis pathogenesis. The study establishes a basis for comprehending the intricate correlation between characteristic genes and Osteoarthritis, as well as for the formulation of individualized therapeutic approaches.

## Introduction

Osteoarthritis (OA), a common long-term joint condition, is widespread globally and is a major contributor to disability, especially in older individuals. Patients experience pain, functional restrictions, and a decrease in their quality of life, which greatly affects their daily activities (1). Factors such as age and obesity are associated with the incidence of OA, which primarily impacts the knee joints, hip joints, hands, and spine. Due to the growing number of elderly individuals and the increasing rates of obesity, the occurrence of OA is progressively on the rise (2). Pain is the primary symptom of OA, which worsens with joint use and improves during rest. Although the pain is typically localized to the affected joint, it can also radiate to other areas. The correlation between pain symptoms and disease severity evaluated through X-ray results remains poorly comprehended (3). In addition to pain, OA leads to functional impairments and limitations in daily activities. Lower limb joint involvement in OA is particularly problematic, leading to reduced mobility among older individuals. For patients in advanced stages of the disease, joint replacement surgery is often necessary to alleviate symptoms and restore function (4, 5). However, this surgery also contributes significantly to the direct medical costs associated with OA. To summarize, OA is a common long-term condition affecting the joints, which has a substantial impact on the well-being and daily existence of those it affects. Despite the progress made in medical interventions, there is presently no viable remedy to decelerate the advancement of the illness.

Multiple signaling pathways, such as Wnt/β-catenin, NF-κB, focal adhesion, HIFs, TGFβ/BMP, and FGF signaling pathways, are involved in OA according to recent studies (6–8). The expression of important downstream factors in articular cartilage cells and synovium is controlled by activated pathways and regulatory factors, which operate through intricate networks of interactions and feedback mechanisms involving Runx2, MMP13, AMPK, PRG4, and others (6). Moreover, studies have indicated that Emodin, a substance that hinders matrix metalloproteinases (MMPs) and a disintegrin and metalloproteinase with thrombospondin motifs (ADAMTS), has the ability to impede the NF-κB and Wnt/β-catenin signaling pathways. As a result, it can improve the deterioration of cartilage in OA (9). Other studies have demonstrated that overexpression of miR-182-5p reduces cartilage cell proliferation by targeting and inhibiting FGF9, leading to increased cell apoptosis rates and inflammatory factors (10). Furthermore, the elimination of UHRF1 boosts the cellular autophagy capability, safeguarding cartilage cells against apoptosis caused by osteoarthritis via the PI3K/AKT/mTOR signaling pathway (11). The results highlight the important functions of particular genes and signaling pathways in the advancement of OA. However, the diagnostic value of many genes in OA has yet to be studied.

Currently, bioinformatics is widely employed in various disease research (12). Integrating relevant bioinformatics studies can more accurately screen differential genes, enabling the exploration of potential disease mechanisms. For this research, we initially obtained OA disease datasets from the Gene Expression Omnibus (GEO) database (13). Then, we performed differential analysis to detect genes that were expressed differently (DEGs) and identified characteristic genes by creating machine learning models. Subsequently, we utilized the expression profiles of these marker genes to group OA samples. Additionally, we examined if there were variances in the prevalence of immune cells among the groups by conducting immune infiltration analysis. To understand the intricate relationships between various genes, we developed the competing endogenous RNA (ceRNA) network for characteristic genes. Finally, we validated the accuracy of our results using external datasets. The aim of this study is to discover new diagnostic genes for OA through bioinformatics, which will establish a theoretical foundation for future personalized and accurate treatment of OA patients.

## Materials and methods

### Data source and preprocessing

We downloaded seven datasets from the GEO database, namely GSE169077 (GPL96 platform, controls: 5, OA: 6), GSE51588 (GPL13497 platform, controls: 10, OA: 40), GSE55235 (GPL96 platform, controls: 10, OA: 10), GSE55457 (GPL96 platform, controls: 10, OA: 10), GSE29746 (GPL4133, controls: 11, OA: 11), GSE117999 (GPL20844, controls: 12, OA: 12), and GSE178557 (GPL13497, controls: 4, OA: 4) (14–16). GSE29746, GSE117999, and GSE178557 were used as a validation dataset. Initially, we employed the R 4.2.2 packages “limma” and “sva” to standardize GSE169077, GSE51588, GSE55235, and GSE55457, and subsequently combine the datasets. The “sva” package in R 4.2.2 utilized the combat function to eliminate batch effects from the combined dataset.

### Identifying differentially expressed genes

The four datasets were merged into a single dataset and batch effects were eliminated using the combat function, we conducted differential analysis on the merged dataset using the “limma” package in R 4.2.2. To identify the DEGs, we set the criteria as |Log FC| ≥ 1 and adjusted *p*-value < 0.05. These criteria were used to determine the significance of gene expression changes between normal and OA samples.

### Construction of machine learning models

We utilized the DEGs to construct two machine learning models for OA: Least Absolute Shrinkage and Selection Operator (LASSO) and Random Forest (RF) (17, 18). The LASSO is employed for the purpose of selecting features and performing regression analysis. It introduces a L1 regularization term to penalize unimportant features, resulting in feature sparsity and model simplification. For our investigation, we utilized the R 4.2.2 and the “glmnet” package to construct the LASSO model. To construct the model, the merged dataset was read, transformed into a feature matrix and target variable, and the “glmnet” function was used. We assessed the effectiveness of the LASSO model by creating visual representations of the LASSO regression path and conducting cross-validation. After analyzing the cross-validation outcomes, we determined the optimal penalty parameter and identified the genes that were chosen as features. RF is an ensemble learning method commonly used for classification and regression tasks. It consists of multiple decision trees, each constructed by bootstrapping the dataset and randomly selecting features. For our study, we utilized the R 4.2.2 software and the “randomForest” package to construct the RF model. The process involved reading the data from the merged dataset, converting it into a feature matrix and target variable, and training the random forest model. After plotting the graph depicting the correlation between the number of trees and error rate, we successfully pinpointed the precise location of the minimum error point. Subsequently, we rebuilt the random forest model and calculated the importance scores for the genes, allowing us to select the significant marker genes. A bubble chart was created using the “ggplot2” package to display the gene importance scores. In order to detect shared disease marker genes between the LASSO and RF models, we utilized the R 4.2.2 package called “VennDiagram.” This facilitated subsequent analysis and comparison of the selected marker genes.

### Analysis of marker genes

In order to observe the patterns of expression of the chosen genes between normal and OA samples, we employed the R 4.2.2 packages “limma” and “ggpubr” to create the violin figure for each gene. The visual depiction facilitates a more instinctive comprehension of the disparities in gene expression between the two groups of samples. Furthermore, we evaluated the discriminatory ability of each gene feature individually by creating receiver operating characteristic (ROC) curves. We used the “pROC” package in R 4.2.2 for this analysis. ROC curves illustrate the sensitivity (true positive rate) vs. the specificity (1 - false positive rate) across different threshold values. The area under the curve (AUC) is computed to measure the capacity of each gene to differentiate between normal samples and OA samples on an individual basis. Greater AUC values indicate superior ability of a specific gene to differentiate between the two groups of samples. Our goal is to enhance our understanding of the potential roles of the selected marker genes in distinguishing between normal samples and OA samples by visually representing their expression patterns and individual discriminatory abilities through the use of create the violin figure and ROC curves.

### Osteoarthritis grouping and differential analysis based on marker genes

By analyzing the median expression levels of marker genes in OA samples (*n* = 66) from the combined dataset, we categorized the OA samples into two groups: the high expression group and the low expression group. We conducted differential analysis between the high and low expression groups using the R 4.2.2 packages “limma” and “pheatmap,” with a with a screening criteria of |Log FC| ≥ 1 and adjusted *p*-value < 0.05. The results were then visualized. Afterwards, we conducted correlation analysis of DEGs using the R 4.2.2 package called “corrplot.” Furthermore, in order to clarify the primary roles of these DEGs, we employed the R 4.2.2 packages called “clusterProfiler,” “org.Hs.eg.db,” and “enrichplot” to conduct Gene Ontology (GO) and Kyoto Encyclopedia of Genes and Genomes (KEGG) analysis on the DEGs.

### Gene set enrichment analysis and gene set variation analysis

In order to further examine the gene expression data and obtain a better understanding of the biological processes and pathways linked to the groups with high and low expression, we utilized two techniques: Gene Set Enrichment Analysis (GSEA) and Gene Set Variation Analysis (GSVA). GSEA is a widely used method for determining whether a predefined gene set is enriched or coupled with the gene expression data. In order to conduct GSEA, we acquired the GSEA program (version 3.0) from the MSigDB website repository and retrieved the subsets “c2.cp.kegg.v7.4.symbols.gmt” and “c5.go.symbols.gmt” from the MSigDB website database (19). These gene sets represent known pathways and biological processes. The GSEA software was utilized to assess the enrichment of these gene sets using the gene expression profiles of the high and low expression groups. The examination was conducted using particular criteria, which consisted of a minimum gene collection of 5, a maximum gene collection of 5,000, resampling 1,000 times, and establishing statistical significance at a *p*-value less than 0.05 and a false discovery rate (FDR) less than 0.25. In contrast, GSVA is a non-parametric technique utilized to evaluate the collective activity levels of gene sets in a specific set of samples. GSVA helps us understand the relative variation of gene sets in samples, revealing functional differences between different sample groups. In order to conduct GSVA enrichment analysis, we acquired the “c2.cp.kegg.v7.4.symbols” and “c5.go.bp.v7.5.1.symbols” files from the database on the MSigDB website. Next, we utilized the R 4.2.2 and the “GSVA” package to compute the enrichment scores for every gene set in each sample, which generated a matrix of enrichment scores. In conclusion, we employed the R 4.2.2 “limma” package to detect dissimilarly expressed pathways and biological functions through comparing the GSVA scores of the high expression and low expression groups. Our objective was to discover and describe the enriched gene sets, pathways, and biological functions linked to the distinct gene expression patterns observed between the high expression and low expression groups.

### Analysis of immune cell correlation with marker genes

In order to examine the association between characteristic genes and immune cells in OA, we employed the CIBERSORT algorithm (20) for the analysis of variations in immune cell infiltration between normal and OA samples. Next, we utilized the Spearman algorithm to conduct correlation analysis in order to investigate the association between immune cells and marker genes. To assess immune function based on ssGSEA, we also used the “limma,” “GSVA,” and “GSEABase” packages in R 4.2.2. We examined the infiltration of immune cells in the marker genes high and low expression groups and presented the findings through box plots.

### Construction of ceRNA network for marker genes

The miRcode database (21) was used to match lncRNA and miRNA. miRTarBase (22), miRDB (23), and TargetScan (24) databases were used to find miRNAs matching marker genes. Interactions between miRNA, lncRNA, and target genes were integrated to construct a ceRNA regulatory network. Cytoscape (version 3.7.2) was used for visualization (25). Finally, three datasets (GSE29746, GSE117999, and GSE178557) were used for validation of the marker genes. The ROC curves of the marker genes in these datasets were constructed using the “pROC” package in R 4.2.2, which was used to validate the accuracy of the marker genes in distinguishing between non-OA and OA patients.

## Results

### Identifying differentially expressed genes

**Figure 1:**
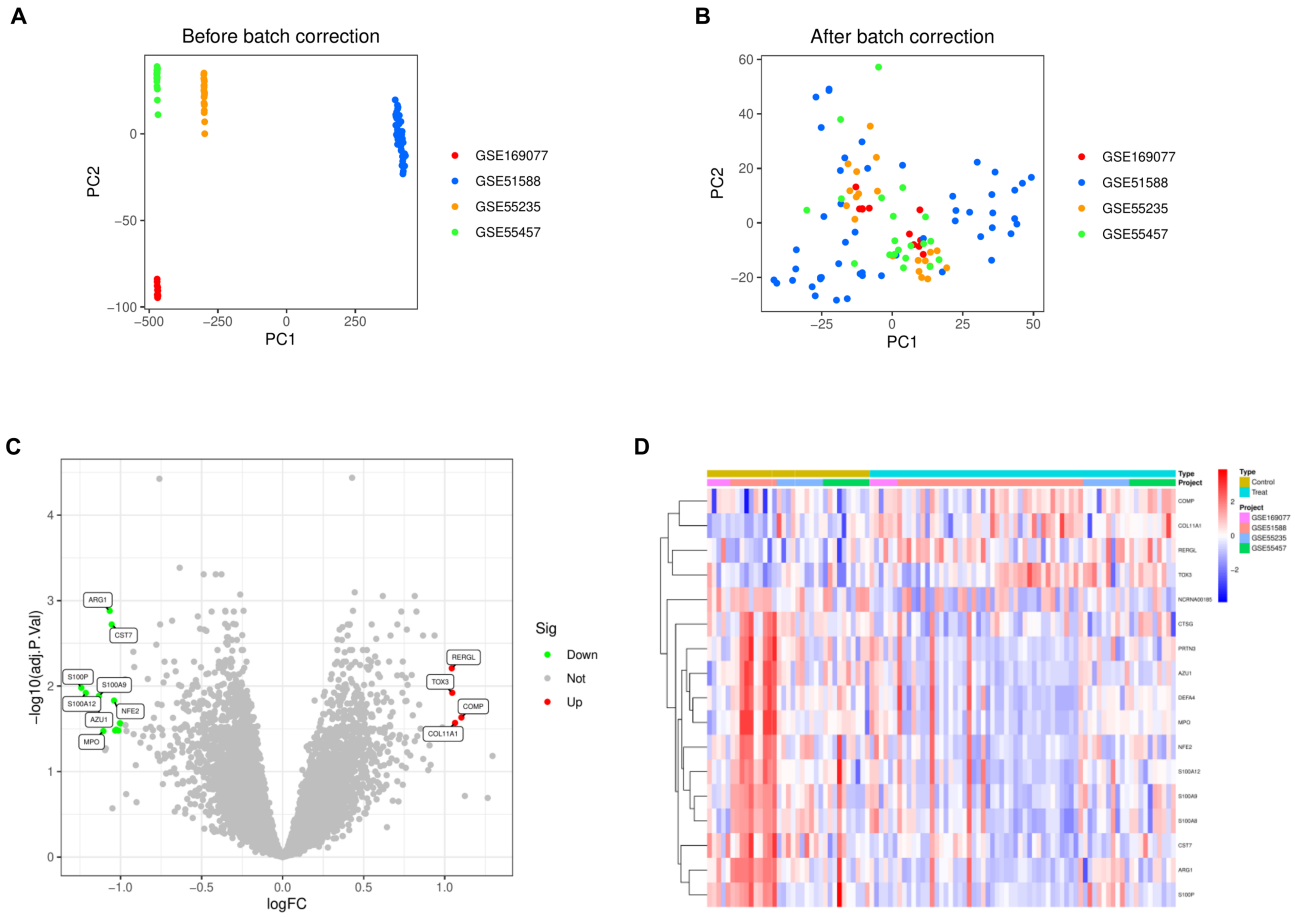
Identification of differentially expressed genes in Osteoarthritis. **(A)** Principal Component Analysis before merging the four data sets. **(B)** Principal Component Analysis after merging the four data sets. **(C)** Volcano map of differentially expressed genes. **(D)** Heat map of differentially expressed genes.

Normalization was conducted on four datasets, namely GSE169077, GSE51588, GSE55235, and GSE55457 (Figures 1A,B). By applying the criteria of |Log FC| ≥ 1 and adjusted *p*-value < 0.05, the differential analysis successfully detected 17 DEGs. These DEGs included 4 genes that were upregulated and 13 genes that were downregulated (Figure 1C). Figure 1D displayed the expression patterns of the identified DEGs through a heatmap.

### Construction of machine learning models

**Figure 2:**
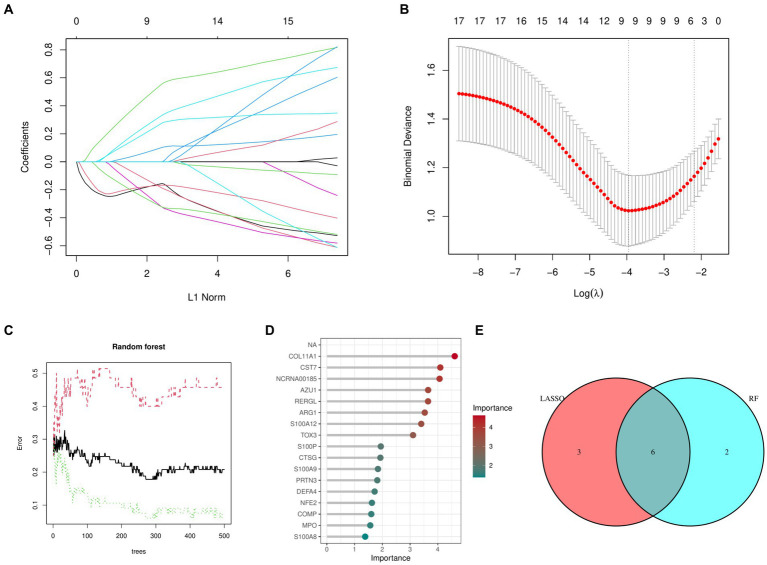
Machine learning models. **(A)** Coefficient diagram of Least Absolute Shrinkage and Selection Operator. **(B)** Parameter plot of Least Absolute Shrinkage and Selection Operator. **(C)** Results of the Random Forest model. **(D)** Importance score of candidate genes for Osteoarthritis. **(E)** Intersectional marker genes for Least Absolute Shrinkage and Selection Operator and Random Forest.

Figures 2A,B shows that the LASSO algorithm in machine learning detected 9 marker genes related to OA, which are ARG1, CST7, RERGL, TOX3, NFE2, COMP, COL11A1, CTSG, and NCRNA00185. In the same way, we used 271 trees, the RF machine learning algorithm discovered a group of 8 marker genes, specifically COL11A1, CST7, NCRNA00185, AZU1, RERGL, ARG1, S100A12, and TOX3 (Figures 2C,D). Figure 2E displays the Venn diagram analysis of the marker genes from both models, uncovering 6 shared genes namely TOX3, ARG1, CST7, RERGL, COL11A1, and NCRNA00185.

### Analysis of marker genes

**Figure 3:**
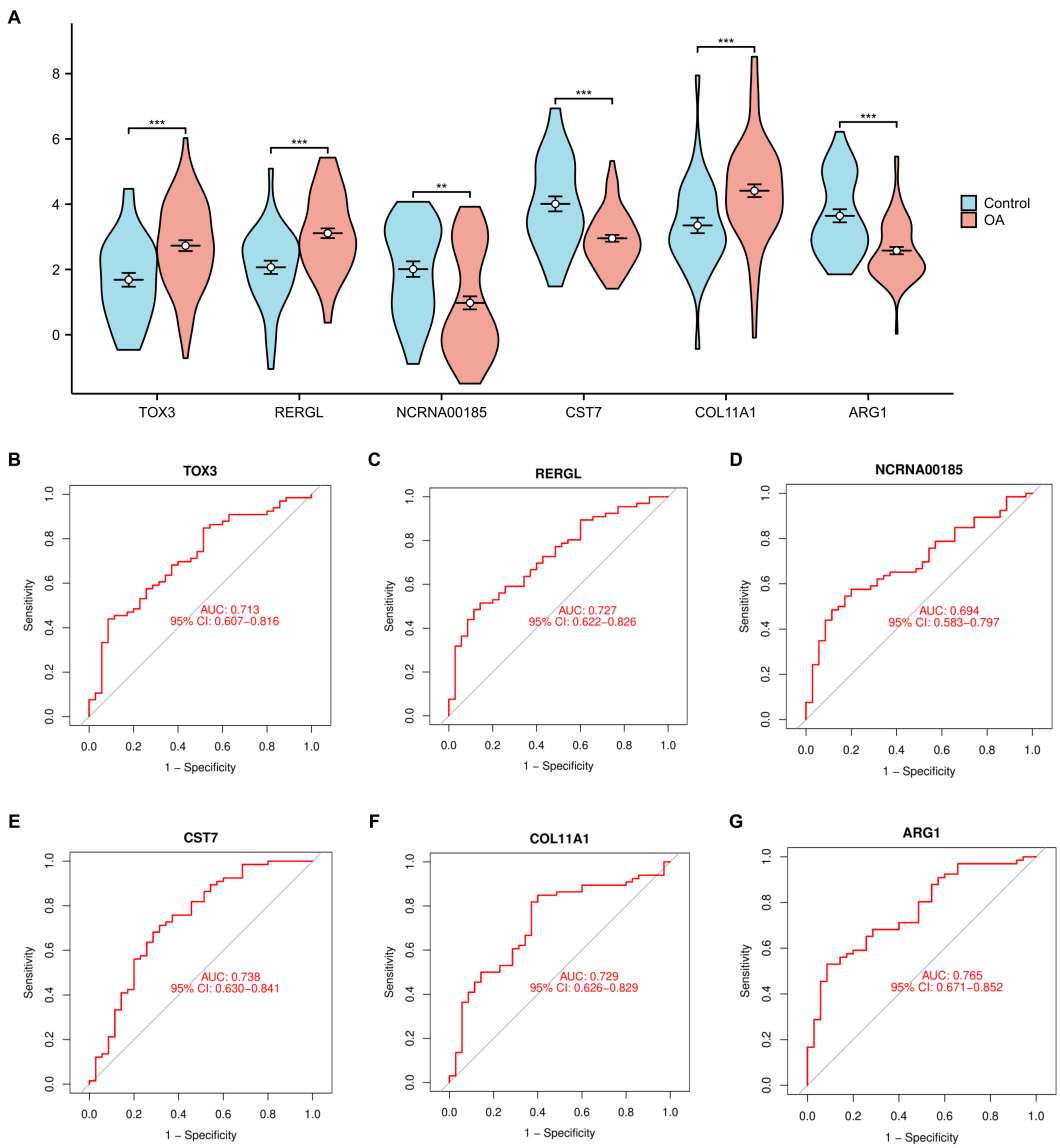
Analysis of marker genes. **(A)** The violin diagram of marker genes. Receiver operating characteristic of TOX3 **(B)**, RERGL **(C)**, NCRNA00185 **(D)**, CST7 **(E)**, COL11A1 **(F)**, and ARG1 **(G)**. *p < 0.05, **p < 0.01, and ***p < 0.001.

In order to offer a more graphical depiction of the 6 overlapping marker genes (TOX3, ARG1, CST7, RERGL, COL11A1, NCRNA00185), a violin figure was generated (Figure 3A). Furthermore, we generated receiver ROC curves for each of the 6 marker genes to evaluate their individual capacity in differentiating normal and OA samples. The findings indicated that TOX3 achieved an AUC value of 0.713, RERGL attained an AUC value of 0.727, NCRNA00185 obtained an AUC value of 0.694, CST7 recorded an AUC value of 0.738, COL11A1 obtained an AUC value of 0.729, and ARG1 exhibited the highest AUC value of 0.765 (Figures 3B–G). The results suggest that the 6 marker genes have a strong ability to differentiate between normal and OA samples. A large number of studies have shown that TOX3 is associated with a wide range of diseases and involves complex biological mechanisms (26, 27). therefore, we selected TOX3 for further study.

### Osteoarthritis grouping and differential analysis based on marker genes

**Figure 4:**
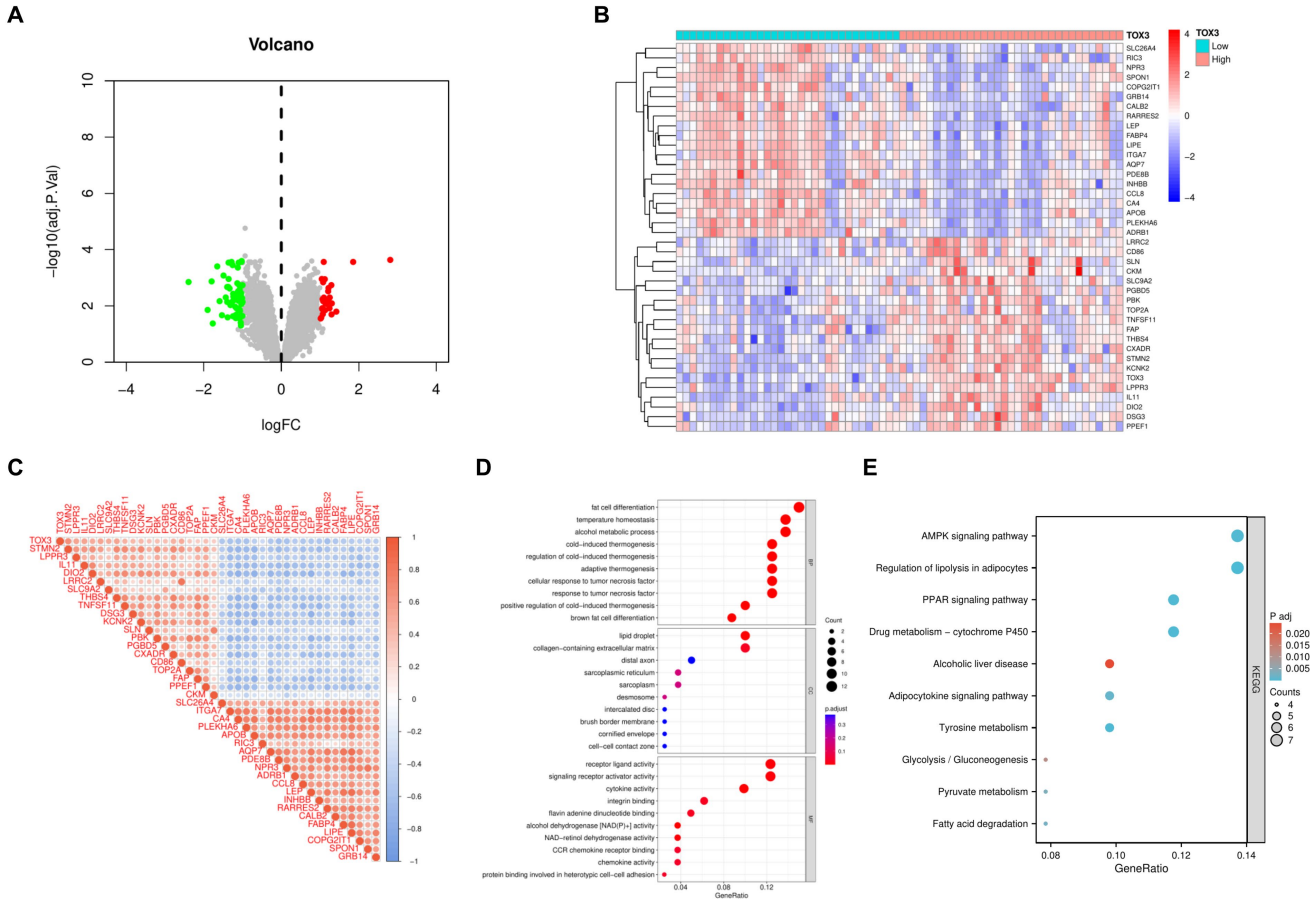
Analysis of high and low TOX3 expression groupings in osteoarthritis. **(A)** Volcano map of differentially expressed genes. **(B)** Heat map of differentially expressed genes. **(C)** Correlation analysis of differentially expressed genes. **(D)** Gene Ontology analysis of differentially expressed genes. **(E)** Kyoto Encyclopedia of Genes and Genomes.

Based on the median expression levels of TOX3 (cutoff value = 2.667), we categorized the 66 OA samples into two groups: a group with high expression (*n* = 32) and a group with low expression (*n* = 34). A comparative analysis was performed on these two groups, using the criteria of |Log FC| ≥ 1 and adjusted *p* < 0.05. The examination uncovered a grand total of 85 DEGs, comprising of 27 genes that were upregulated and 58 genes that were downregulated (Figure 4A). To visualize the expression patterns of the top 20 upregulated and downregulated DEGs (Figure 4B), a heatmap was created. Additional correlation analysis of DEGs revealed a significant positive correlation between TOX3 and STMN2, LPPR3, and IL11. Conversely, a notable negative correlation was detected between TOX3 and GRB14, SPON1, and COPG2IT1. These findings suggest that TOX3 is influenced by intricate regulatory mechanisms in OA, as depicted in Figure 4C. The DEGs underwent GO and KEGG analyses. The analysis of GO showed that in biological processes (BP), DEGs were primarily enriched in adipocyte differentiation, thermoregulation, and alcohol metabolism. DEGs in cellular components (CC) were mainly linked to lipid droplet, extracellular matrix containing collagen, and distal axon. DEGs in molecular functions (MF) primarily participated in receptor ligand activity, activation of signaling receptors, and cytokine activity (Figure 4D). According to the KEGG analysis, DEGs showed significant enrichment in the AMPK signaling pathway, adipocyte lipolysis regulation, the PPAR signaling pathway, and various related pathways (Figure 4E). The results indicate that the breakdown of fat could have a substantial impact on the development and advancement of OA.

### Gene set enrichment analysis and gene set variation analysis

**Figure 5:**
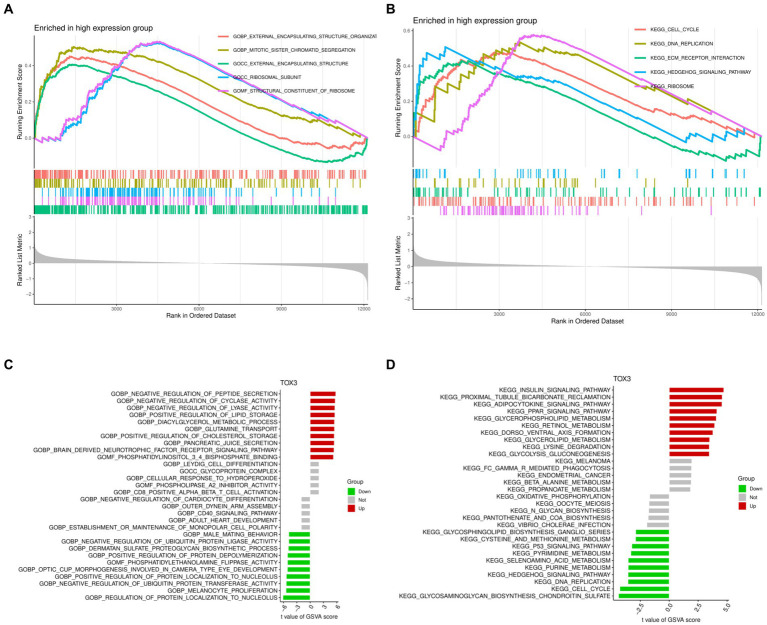
Gene Set Enrichment Analysis and Gene Set Variation Analysis. **(A)** Gene Ontology of Gene Set Enrichment Analysis in high expression group. **(B)** Kyoto Encyclopedia of Genes and Genomes of Gene Set Enrichment Analysis in high expression group. **(C)** Gene Ontology of Gene Set Variation Analysis. **(D)** Kyoto Encyclopedia of Genes and Genomes of Gene Set Variation Analysis.

GSEA and GSVA were performed to investigate the functional disparities associated with DEGs between the TOX3 high-expressing cohort and the low-expressing cohort. The GSEA findings indicated that the TOX3 high-expression group exhibited notable enrichment in the organization of external encapsulating structures, segregation of mitotic sister chromatids, and functions related to external encapsulating structures (Figure 5A). Furthermore, the analysis of pathway enrichment revealed enrichment in the cell cycle, interaction with ECM receptors, the hedgehog signaling pathway, and various other signaling pathways (Figure 5B). The GSVA analysis revealed that in the high-expression group of TOX3, pathways associated with the promotion of lipid storage, diacylglycerol metabolism, and cholesterol storage were found to be upregulated. On the other hand, in the group with low expression of TOX3, the upregulation of pathways related to phosphatidylethanolamine flippase function, biosynthesis of dermatan sulfate proteoglycan, and inhibition of ubiquitin protein ligase activity was observed (Figure 5C). Furthermore, the TOX3 high-expression group exhibited significant enhancement in various pathways, including but not limited to the insulin signaling pathway, adipocytokine signaling pathway, glycerophospholipid metabolism, and PPAR signaling pathway, as revealed by pathway enrichment analysis. In contrast, the group with low expression of TOX3 showed significant enhancement in pathways such as chondroitin sulfate biosynthesis of glycosaminoglycan, cell cycle, and the p53 signaling pathway (Figure 5D). The results suggest notable variations in functionality between the high-expression group of TOX3 and the low-expression group. The group with high expression of TOX3 seems to have a strong connection with lipid metabolism, whereas the group with low expression of TOX3 may be more linked to cell proliferation.

### Immune cell correlation analysis of marker genes

**Figure 6:**
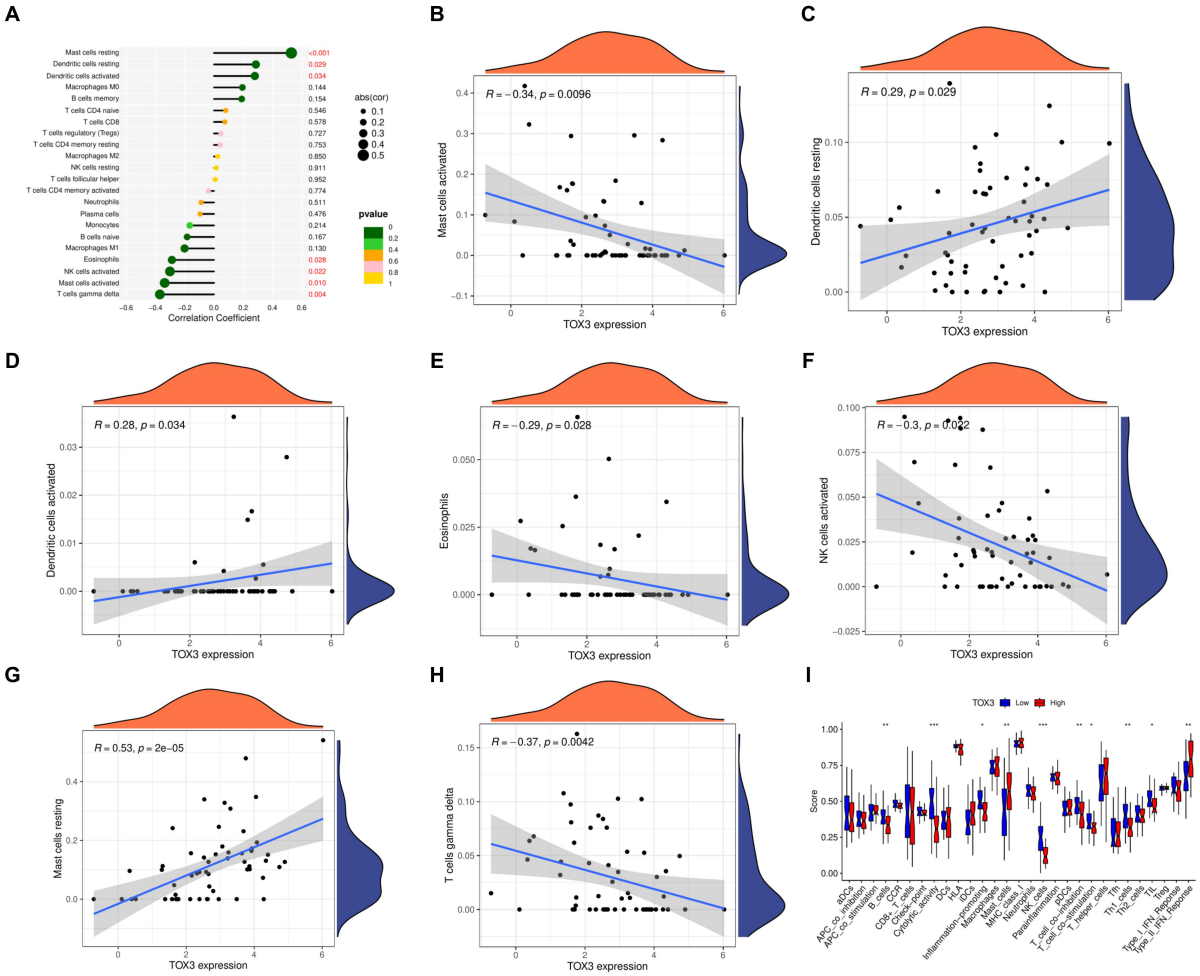
Immunological correlation analysis. **(A)** A landscape wide map of TOX3 correlation with all immune cells. **(B)** Correlation of TOX3 and Mast cell activation. **(C)** Correlation of TOX3 and Dendritic cells resting. **(D)** Correlation of TOX3 and Dendritic cells activated. **(E)** Correlation of TOX3 and Eosinophils. **(F)** Correlation of TOX3 and NK cells activated. **(G)** Correlation of TOX3 and Mast cells resting. **(H)** Correlation of TOX3 and T cells gamma delta. **(I)** Immune cell infiltration analysis of TOX3 high and low expression groups. *p < 0.05, **p < 0.01, and ***p < 0.001.

We utilized the CIBERSORT algorithm to conduct immune scoring for each sample using the combined dataset. Next, the Spearman algorithm was utilized to perform correlation analysis on immune cells and TOX3. The results showed that TOX3 had a correlation with a variety of immune cells. TOX3 displayed a less powerful negative correlation with Mast cells activated, Eosinophils, NK cells activated, and T cells gamma delta. In contrast, TOX3 exhibited a less powerful positive correlation with Dendritic cells resting and Dendritic cells activated. TOX3 had some positive correlation with Mast cells resting (Figures 6A–H). These results indicated that TOX3 correlates with a wide range of immune cells, and although these correlations were not strong, they also suggested that TOX3 may play a crucial role in the complex immune processes associated with OA. Moreover, we investigated if there are any notable variations in the immune system between the high-expression and low-expression groups of TOX3. Distinct immune profiles were revealed through an analysis of immune cell infiltration conducted on the two groups. The TOX3 high-expression group showed a notable increase in Mast cells and Type II IFN Response, in contrast to the TOX3 low-expression group. In contrast, the TOX3 high-expression group showed significant downregulation of B cells, cytolytic activity, inflammation-promoting cells, NK cells, pDCs, T cell co-inhibition, Th1 cells, and Th2 cells (Figure 6I). The findings suggested notable variations in the composition of immune cells between the high-expression and low-expression groups of TOX3. Collectively, these results indicated that TOX3 was not only associated with particular types of immune cells but also played a role in the immune variations observed between the high-expression and low-expression groups of OA. The findings provided insight into the possible role of TOX3 in the immune regulation of OA.

### Construction of ceRNA network for marker genes

**Figure 7:**
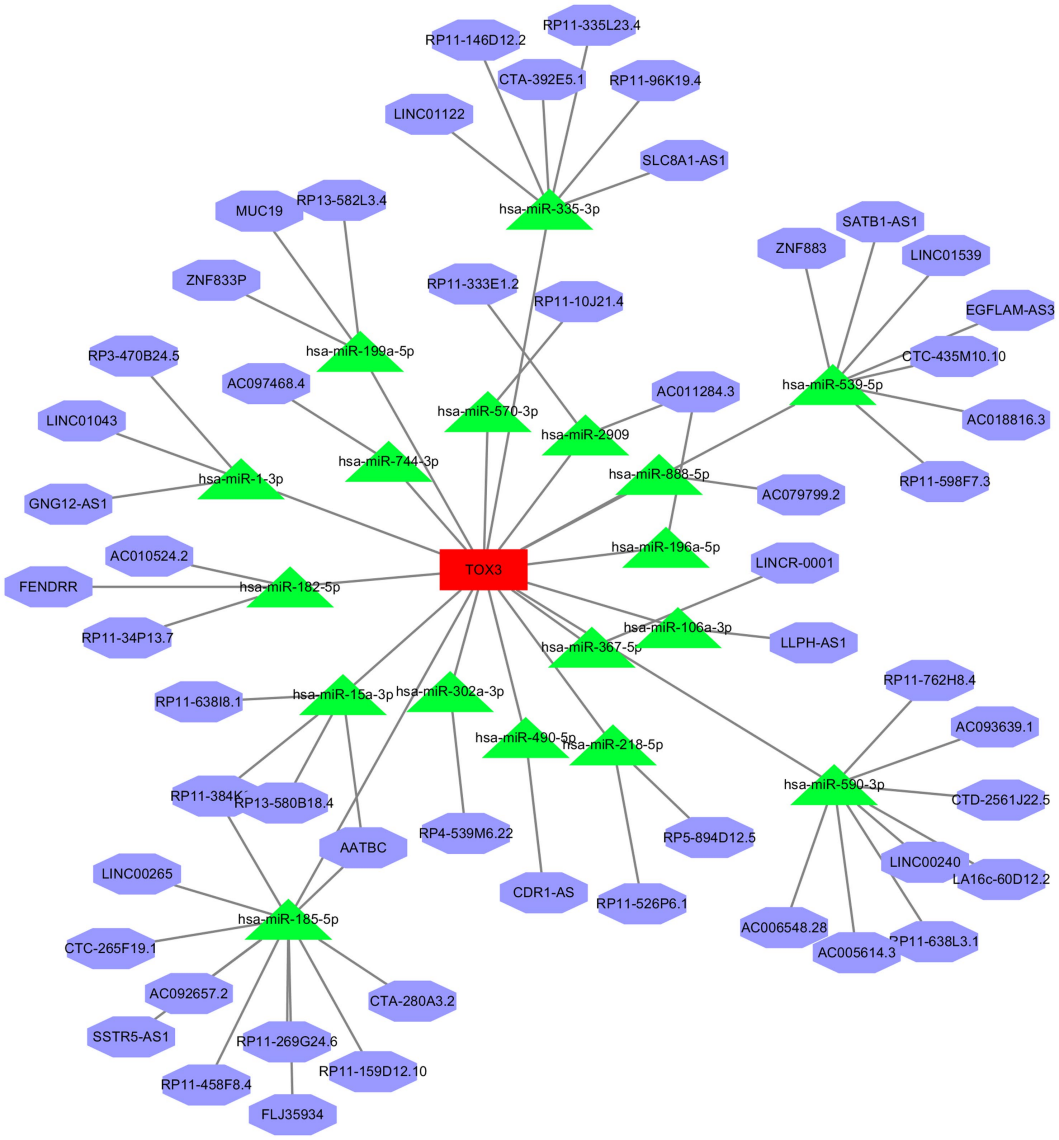
Construction of ceRNA networks. The red is the mRNA, TOX3. The green is miRNAs. The purple is lncRNAs.

**Figure 8:**
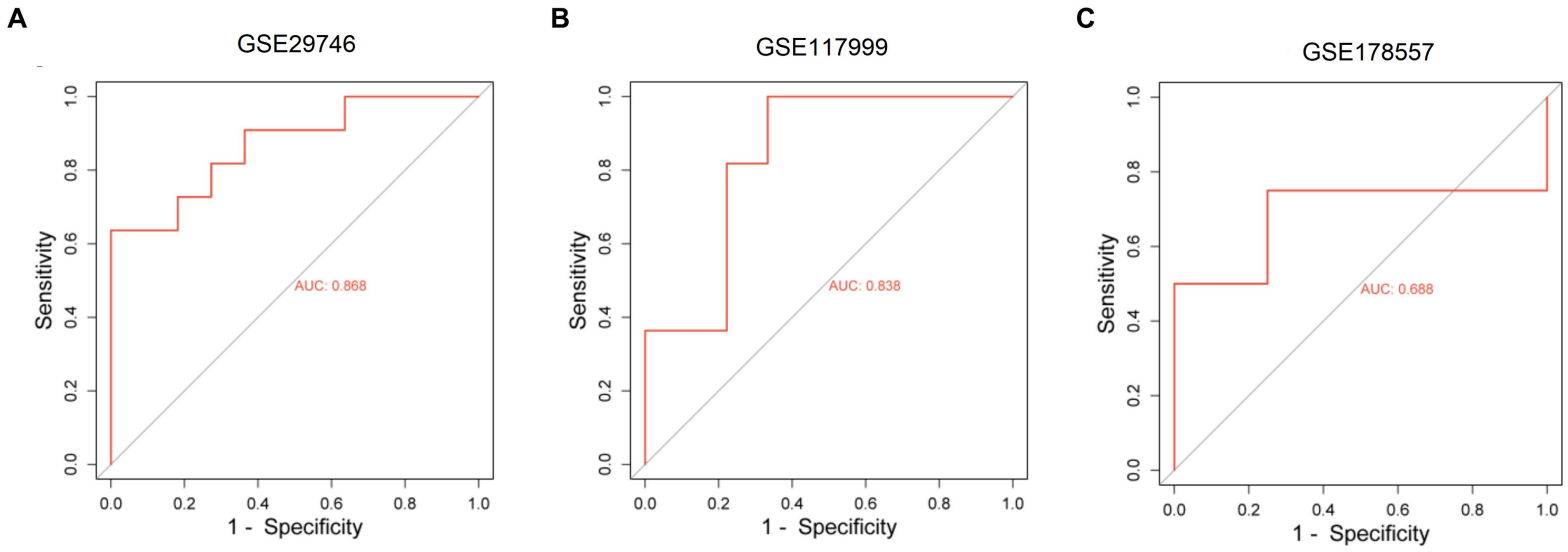
Receiver operating characteristic of external independent validation datasets. Receiver operating characteristic of GSE29746 **(A)**, GSE117999 **(B)**, and GSE178557 **(C)**.

We constructed a ceRNA network for TOX3, which revealed 57 lncRNAs and 18 miRNAs involved in 57 lncRNA-miRNA interactions. Furthermore, TOX3 was discovered to interact with 18 miRNAs, resulting in the formation of 18 miRNA-mRNA interactions. Firstly, we observed interactions between the TOX3 gene and multiple miRNAs, which play pivotal roles in the pathophysiological processes of OA. For instance, the interaction of miR-185-5p with TOX3 has been reported to be associated with OA (28). TOX3 may exert its influence by binding to these miRNAs, thereby affecting their regulation of other mRNAs and consequently impacting the progression of OA. Secondly, we identified interactions between various lncRNAs and miRNAs, where these lncRNAs may serve as “sponges” for miRNAs, attenuating their regulation of other target genes. For example, interactions such as RP5-894D12.5 with miR-218-5p and LINC01043 with miR-1-3p are likely to play a role in the regulation of OA. Additionally, some miRNAs simultaneously interact with multiple lncRNAs and mRNAs, suggesting their extensive regulatory roles in OA. This multi-pathway regulatory mechanism may have complex implications for disease progression. For instance, miR-185-5p, in addition to its interaction with TOX3, also engages with several other lncRNAs and mRNAs, potentially affecting the gene expression related to OA through these interactions (Figure 7). Finally, we validated the diagnostic value of TOX3 using ROC curves based on three external independent datasets, GSE29746, GSE117999 and GSE178557. The ROC of GSE29746 was 0.868 (Figure 8A), the ROC of GSE117999 was 0.838 (Figure 8B) and the ROC of GSE178557 was 0.688 (Figure 8C). This validation supports the accuracy and reliability of TOX3 as a potential feature gene for diagnosing OA. Our research findings collectively suggest that TOX3 shows potential as a new marker gene that could aid in the detection of OA. The ceRNA network involving TOX3, along with its validated expression pattern, further supports its potential significance in the context of OA.

## Discussion

OA is a common long-term condition affecting people globally and has a substantial impact on their quality of life. This condition is marked by the gradual deterioration of joint cartilage, resulting in discomfort, swelling, and limited joint movement (29). The disabling characteristics of OA result in occupational impairment and diminished quality of life for those impacted. Recent studies have brought attention to the role of inflammation and immune reactions in the development of OA. Abnormal activation of these processes can contribute to cartilage damage and joint dysfunction (30, 31). Obesity, a known risk factor for OA, exacerbates joint deterioration by increasing mechanical stress on the joints and releasing inflammatory substances from adipose tissue (32, 33). Over the past few years, numerous functional genes have been recognized as crucial contributors to the advancement and growth of OA. Examples include GREM1, FRZB, DKK1, BCL2, and BAX (34). It is crucial to comprehend the diagnostic significance of these genes in OA for enhancing early identification and formulating focused therapeutic approaches. Moreover, the examination of numerous genes in OA for their diagnostic significance remains unexplored. In general, additional investigation is required to clarify the exact roles of these genes in the development of OA and their potential for diagnosis and treatment.

The main focus of this study was on OA, with the objective of identifying DEGs that are linked to this disease. We utilized four datasets, namely GSE169077, GSE51588, GSE55235, and GSE55457, and applied strict criteria to screen for DEGs. By employing machine learning algorithms, particularly LASSO and RF, we have discovered a group of marker genes. Among these genes, TOX3, ARG1, CST7, RERGL, COL11A1, and NCRNA00185 were found to be shared by both models. In order to acquire a more profound comprehension of the function of these marker genes, we carried out additional investigations. The ROC curve analysis exhibited the capacity of these genes to accurately differentiate normal samples from OA samples. Specifically, our attention was directed toward the TOX3 gene, and we categorized the OA samples into two groups, high and low expression, depending on the levels of TOX3 expression. By conducting differential analysis and functional enrichment analysis, we discovered DEGs linked to lipid metabolism, indicating their potential role in the development and advancement of OA. In addition, we conducted GSEA and GSVA investigations to examine functional disparities between the TOX3 high expression cohort and low expression cohort. The analyses uncovered connections with lipid metabolism in the group with high expression and cell growth in the group with low expression. Furthermore, we examined the association between TOX3 and immune cells utilizing the CIBERSORT algorithm, and we detected notable disparities in the infiltration of immune cells among the two groups with varying TOX3 expression. According to previous studies, TOX3 was mainly involved in the regulation of estrogen receptor signaling. Combined with the results of the present study, we hypothesized that through the hormonal pathway, estrogen levels may indirectly affect lipid metabolism and thus have a link with TOX3 (35). Second, lipid metabolism plays a key role in the function of immune cells. Lipids are important components of cell membranes, and immune cells need to regulate their lipid metabolism to perform various functions such as phagocytosis, cytokine production, and immune signaling (36, 37). At the same time, lipid metabolism can also affect the inflammatory response, which is closely related to immune cell function. Disorders of lipid metabolism may lead to inflammation, which in turn affects the immune response in a variety of contexts, including OA (38). Thus, TOX3, there may be a complex link between lipid metabolism and immune cells that influences the development of OA. Moreover, a network of ceRNAs was established using TOX3, uncovering intricate connections involving numerous lncRNAs, miRNAs, and TOX3. The TOX3 regulatory mechanisms in OA are illuminated by this network, offering valuable insights. To validate our findings, we verified the diagnostic value of TOX3 using ROC curves based on three external independent datasets (GSE29746: AUC = 0.868, GSE117999: AUC = 0.838, and GSE178557: AUC = 0.688). All ROC results showed that TOX3 has excellent diagnostic value, which was in consistent with our previous findings. In the context of OA, the significance of these identified genes, especially TOX3, is emphasized by our study. The results of this study enhance our comprehension of the molecular processes involved in the progression of OA and could potentially impact the diagnosis and management of this condition.

TOX3 is a crucial gene that encodes proteins and has diverse functions in cellular processes. The high mobility group box domain of this protein is engaged in interactions with CREB protein and CITED1 (35). The neuroprotective effects of TOX3 have been discovered through its ability to prevent neuronal cell death caused by endoplasmic reticulum stress or excessive BAX expression. This is accomplished by regulating the expression of genes that prevent cell death and genes that promote cell death (39). Furthermore, TOX3 plays a role in controlling the transcription linked to estrogen response elements and estrogen response promoters (35). It is worth mentioning that TOX3 has been linked to studies on cancer (40, 41). A decrease in TOX3 expression has been noted in cases of acute myeloid leukemia (AML). Nevertheless, increased TOX3 expression in individuals with AML is linked to inferior survival results, suggesting its potential as a biomarker for AML (42). Although TOX3 has been implicated in various types of cancer, there is currently a scarcity of *in vivo* and *in vitro* experimental studies investigating the association between TOX3 and OA. In this study, a new marker gene for OA, namely TOX3, was identified through a comprehensive bioinformatics approach. After further analysis of the expression pattern and mechanism of function, etc., TOX3 was found to play an important role in OA. These findings provided new directions and potential targets for the diagnosis and treatment of OA, and are important for the development of TOX3 in the field of OA understanding and research.

Certainly, although this study offers valuable perspectives, it is important to recognize certain constraints. A constraint of this study is that all the samples from OA patients utilized were acquired from publicly available databases. Therefore, further validation of the clinical value of TOX3 in OA would require extensive recruitment of clinical patients in future studies. Furthermore, a thorough comprehension of the complex mechanisms and associations of TOX3, lipid metabolism, and the role of immune cells in OA requires extensive *in vivo* and *in vitro* experiments. These experiments could offer a more comprehensive understanding of the functional significance of TOX3, lipid metabolism, and the role of immune cells in the development of OA. Future studies that focus on these constraints have the potential to improve our comprehension of TOX3 and its viability as a target for diagnosis and treatment in OA.

## Conclusion

This study utilized OA datasets to identify 17 DEGs associated with OA. By employing LASSO and RF machine learning models, six common marker genes, including TOX3, were identified. Further analysis demonstrated that TOX3 exhibits remarkable performance in distinguishing between normal and OA samples, and it may be implicated in lipid metabolism and immune cell infiltration. These findings offer novel insights into the mechanisms underlying the development and progression of OA, and they pave the way for the development of innovative diagnostic and therapeutic approaches in the future.

## Data availability statement

The original contributions presented in the study are included in the article/supplementary material, further inquiries can be directed to the corresponding authors.

## Ethics statement

Ethical approval was not required for the study involving humans in accordance with the local legislation and institutional requirements. Written informed consent to participate in this study was not required from the participants or the participants’ legal guardians/next of kin in accordance with the national legislation and the institutional requirements.

## Author contributions

ZW: Conceptualization, Methodology, Visualization, Writing – original draft. SD: Investigation, Methodology, Writing – original draft. CZ: Formal analysis, Resources, Writing - review & editing. HZ: Methodology, Writing – original draft. YL: Investigation, Software, Writing – review & editing. JY: Data curation, Methodology, Writing – original draft. YJ: Investigation, Methodology, Validation, Writing – original draft. XW: Data curation, Investigation, Validation, Writing – review & editing. YW: Conceptualization, Formal analysis, Resources, Visualization, Writing – original draft.
